# Targeting Investment in On-farm Surface Water Storage for Groundwater Conservation

**DOI:** 10.1007/s00267-025-02363-3

**Published:** 2026-01-09

**Authors:** Kent F. Kovacs

**Affiliations:** Department of Accounting, Economics, and Finance, University of Arkansas at Little Rock, Little Rock, AR USA

## Abstract

Optimal investment in conservation requires balancing the benefits of conservation against its costs. We model the benefit of groundwater, less the cost of conserving groundwater through a subsidy for on-farm surface water storage. On-farm surface water storage reduces arable land but provides a substitute for groundwater used in irrigation. The average social net benefit from conserving water is $12.32 per acre-foot after thirty years with the current subsidy for surface storage, and the aquifer thickness rises by 10.6%. The average social net benefit of groundwater conserved rises by lowering the subsidy, but the volume of conservation also declines. A third of the sites where groundwater is nearly exhausted after thirty years increase surface storage with the subsidy and experience a rebound in groundwater volumes. Conserving groundwater with the subsidy generates the highest net benefit for sites with a high yield for rice, a low yield for dryland soybean, a low depth to the aquifer, and high natural recharge.

## **Introduction**

The decline of the groundwater table from irrigation and the outflow of water from surface water bodies into the ground can harm the environment (Margat and van der Gun 2013). The demand for groundwater is expected to grow as the temporal variability in surface water flows increases (Taylor et al. 2013). Reduction of groundwater withdrawals is possible with more efficient irrigation practices, surface water storage and transport, non-irrigated cropping or land fallowing, managed aquifer recharge, and drought-tolerant crop varieties (NRC 1997). Some regions have enough rainfall to support agriculture, but this rain comes at a time other than the growing season. The storage and transport of surface water for use in the growing season is possible through public or private infrastructure. On-farm surface water storage removes valuable arable land but lowers the cost of irrigation and may conserve a scarce aquifer resource. We develop an economic model to determine the benefit and cost of conserving groundwater through subsidies (specifically, tax credits) for on-farm surface storage investment.

Understanding the net benefit of conserving groundwater requires knowledge of the discounted sum of the present value of future benefits and costs of conserving groundwater through subsidies. The benefit of conservation for an agricultural landowner is lower future extraction costs and greater certainty of groundwater for irrigation if there is drought. The value of lower future extraction costs depends on spatially variable hydro-agricultural conditions like the depth to groundwater, natural recharge, and the yield of crops, both irrigated and non-irrigated.

The subsidies represent a cost to taxpayers, but the greater future profits to agriculture from the less expensive irrigation due to surface water storage mitigate the social cost. The conservation cost per unit of groundwater depends on how effectively surface storage conserves groundwater relative to other options, like growing dryland crops. Spatial variation is also present in the cost of conserving groundwater with a subsidy. Only by putting the benefit and cost of conserving groundwater together can we determine the net benefit of a conservation investment at a particular location. This may help the managers of state and federal programs prioritize where to target scarce funds for subsidies for surface storage.

No prior study evaluates through economic modeling how agricultural site characteristics affect the net benefit of conserving groundwater for a range of subsidies on surface storage to guide state and federal program priorities. Conservation program managers who allocate a limited amount of funds for groundwater conservation naturally would want to subsidize surface storage where this achieves the highest net benefits for agriculture. The criterion of net benefit is a useful metric to guide conservation, but this requires a consistent methodology to determine both the benefit and cost sides. Our conceptual framework and the development of the economic model determine site-level net benefits to evaluate current and alternative subsidies for storage and better target locations for subsidy support through site-level characteristics.

In terms of broader contributions, this study adds to the international literature by providing long-term, spatially explicit evidence on how subsidy design affects both the costs and benefits of groundwater conservation. While much prior research has emphasized short-term adoption or aggregate impacts (Varady et al. 2013; Giordano 2009), our results highlight the dynamic accumulation of benefits and the uneven site-level responses that shape overall outcomes. The findings suggest that tailoring subsidy intensity to local crop and aquifer conditions could improve both the efficiency and effectiveness of groundwater conservation subsidies worldwide.

Our model application is to the Lower Mississippi River Basin of Arkansas, where the alluvial aquifer is the third most heavily used in the United States and critical for rice production in the country (Konikow 2013). The Mississippi Embayment contains several aquifer layers, and the topmost layer is the unconfined alluvial aquifer called the Mississippi River Valley Alluvial Aquifer (MRVA). The use of on-farm reservoirs and tailwater recovery systems to address the challenges of groundwater overdraft and water quality damage from agricultural runoff in Arkansas has been studied for decades (Hristovska et al. 2010). Popp et al. (2003) examine how reservoirs impact profitability, water use, and sediment control for rice-soybean farms in Eastern Arkansas. Hill et al. (2006) compare alternative water conservation investments in the Grand Prairie Region of Arkansas, including on-farm reservoir cost-share and river water diversion, and the cost-share alternative is found to be the most effective.

A related body of literature focuses on optimization-based and GIS-driven spatial targeting of agricultural water storage and recharge practices, even when on-farm reservoirs are not the sole focus. These studies commonly frame location decisions as optimization problems—often multi-objective—balancing farm profits, groundwater sustainability, and downstream impacts (Kumari and Pathania 2026; Naseri et al. 2021). Complementing these approaches, GIS-based site selection and multicriteria decision analysis (MCDA) studies identify optimal locations for agricultural reservoirs or water harvesting structures using spatial indicators such as slope, drainage density, soil type, runoff potential, and proximity to farms or conveyance networks (Dziuba et al. 2025; Al-Khuzaie et al. 2020). While GIS/MCDA approaches typically emphasize physical suitability rather than economic optimization, together these literatures demonstrate how spatially explicit data and optimization frameworks can be integrated to target on-farm water storage investments toward locations where they maximize hydrologic effectiveness and economic efficiency at field to watershed scales.

Several recent studies consider the benefit of conserving water in the MRVA. Kovacs and Rider (2023) use the hedonic property price model with the sale of agricultural land to estimate the value of alluvial groundwater in Arkansas. The spatial variation in the value of groundwater based on the hedonic model is significant, ranging from $0 to $78 per acre-foot, but the average value across the study area is $9 per acre-foot. Kovacs and Durand-Morat (2020) use an optimization model to examine how the hydrologic property of lateral flows can influence the value of groundwater in the MRVA. Portions of the aquifer with larger hydro-conductivity provide less benefit ($23.68 per acre-foot) than portions of the aquifer with limited lateral flows ($33.06 per acre-foot).

The social cost of conserving groundwater with subsidies for surface storage has been considered for some watersheds in Arkansas, but never for the entire MRVA in the state (Kovacs et al. 2014; Kovacs et al. 2015). Tran et al. (2019) consider the relevance of crop mix change in response to water policy for managed aquifer recharge and surface storage in Arkansas. Agricultural landowners have a willingness to pay to convert land into surface storage of more than $5,279 per acre in water-scarce regions of Arkansas (Kovacs et al. 2019). The return on investment in surface storage to landowners over thirty years ranges from 4% to 21% depending on the assumptions for off-season rainfall and the cost of construction (Kovacs and Mancini 2017).

## Study Region

The withdrawal of groundwater from the MRVA can exceed natural recharge, and depressions in the groundwater table form where irrigation is prominent. The saturation of the alluvial can be less than 30% of pre-settlement (ADA DNR 2023). Both federal and state programs offer tax credits for the construction of surface water storage. However, there is limited understanding of the net benefits to society from groundwater conservation with these subsidies.

To address these challenges, both federal and state programs provide financial assistance for the construction of on-farm surface water storage and tailwater recovery systems, which can reduce pressure on the aquifer. The US Department of Agriculture (USDA) supports on-farm water storage in Arkansas primarily through Natural Resource Conservation Service (NRCS) programs such as Environmental Quality Incentives Program (EQIP), which cost-shares conservation practices, including irrigation reservoirs and tailwater recovery systems, when they address concerns like aquifer depletion, as well as through the Conservation Stewardship Program (CSP) and Regional Conservation Partnership Program (RCPP), which support broader stewardship and watershed-scale water initiatives (USDA-NRCS 2025). While groundwater conservation is not always the explicit purpose of these federal programs, many funded practices reduce groundwater pumping by increasing access to stored surface water. Eligibility generally requires that applicants be agricultural producers with eligible land, and applications are ranked according to environmental benefits and conservation priorities established at the state and national levels.

At the state level, Arkansas also incentivizes on-farm water storage through programs such as the Groundwater Conservation Tax Credit, which directly encourages the construction of reservoirs and other surface-water systems to reduce groundwater withdrawals, and the Agricultural Water Quality Loan Program, which provides low-interest financing for similar infrastructure (ADA DNR 2023). These state programs aim to shift irrigation demand away from declining aquifers, and eligibility is typically based on land ownership, conservation district approval, and compliance with state technical design standards. Despite these national and state investments, there remains limited evidence on how such programs affect the net benefits to society from groundwater conservation. Understanding where subsidies generate the highest returns is essential for improving the efficiency and targeting of conservation programs.

## Model

We start with the conceptual framework for quantifying the benefit of an additional unit of water in an aquifer, and then for finding the cost of an additional unit of water underground using subsidies for surface storage. Next, we explain the empirical model components, such as the land and water resource decisions, that inform the constraints on the agriculture profit maximization objective. The results from the solution to the maximization problem have the elements to construct the net benefit of conserved water based on the conceptual framework.

### Conceptual Framework

Society receives at time *t*, a profit, $P(s(t),c(t))$, in an agricultural region from its stock of capital, both the natural kind such as the aquifer, $s(t)$, and all other capital (e.g. tools and knowledge), $c(t)$. The aquifer provides non-market benefits (e.g. avoidance of subsidence), but our model only maximizes the flow of profit from crops due to limited information about the non-market benefits. The net present value of profit (Eq. 1) from agriculture at time *t* is

1 $$V(t)={\int }_{t}^{\infty }{e}^{r(\tau -t)}P(s(\tau ),c(\tau ))d\tau ,$$

where the discount factor ${e}^{r(\tau -t)}$ puts the flow of profits over the infinite planning horizon into period *t* values. Maler et al. (2008) defines the benefit of a unit of groundwater in an aquifer as

2 $$b(s(t))\equiv \frac{\partial V(t)}{\partial s(t)}.$$

The definition of the benefit of a unit of water in the aquifer in Eq. (2) is the added present value of profits society receives in perpetuity from a marginal increase in groundwater. As a consequence, the value of a unit of groundwater depends on agricultural markets, the physical attributes of the aquifer, irrigation technology and infrastructure (e.g. on-farm surface storage and irrigation practices), government support and conservation programs (e.g. subsidies), water resource institutions, and the choice of the discount rate.

The social cost of conserving a unit of groundwater depends on how much social cost and groundwater conservation the subsidies for surface storage induce. The present value of taxes paid to encourage surface storage is $PT(t)={\int }_{0}^{t}{e}^{r\tau }T(\tau )d\tau$, where $T(\tau )$ indicates the higher taxes paid at time $\tau$. The present value of profits to agriculture with the subsidies is ${V}^{{\prime} }(t)={\int }_{t}^{\infty }{e}^{r(\tau -t)}P({s}^{{\prime} }(\tau ),{c}^{{\prime} }(\tau ))d\tau$ and then without the subsidies is $\hat{V}(t)={\int }_{t}^{\infty }{e}^{r(\tau -t)}P(\hat{s}(\tau ),\hat{c}(\tau ))d\tau$.

The social cost of the subsidies at time *t* are the present value of the extra taxes for the public less the change in the present value of profits to agriculture with versus without the subsidies, $PT(t)-({V}^{{\prime} }(t)-\hat{V}(t))$. The cost per unit of groundwater in an aquifer conserved with subsidies for surface storage (Eq. 3) is

3 $$c(s(t))=\frac{PT(t)-({V}^{{\prime} }(t)-\hat{V}(t))}{({s}^{{\prime} }(t)-\hat{s}(t))},$$

where $({s}^{{\prime} }(t)-\hat{s}(t))$ is the difference in the aquifer stock with versus without the subsidies in time *t*.

The net benefit of a unit of groundwater conserved in the aquifer due to subsides for on-farm surface storage (Eq. 4) is

4 $$nb(s(t))=b(s(t))-c(s(t)).$$

The average of the net benefits across all sites on the landscape is an indicator of whether the subsidies program is generating societal gains overall, but there are also site-specific net benefits that reveal where on the landscape the program is most successful or harmful.

### Empirical Model of Agricultural Profit Maximization

The model maximizes the present value of agricultural profits across sites and periods under scenarios with and without subsidies for surface storage. The optimization is simultaneous across all sites, which assumes cooperative rather than competitive decision-making. Both cooperative and competitive irrigation occur in practice. Evidence from Gisser and Sanchez (1980) suggests that the difference in pumping outcomes under the two assumptions is often small, but later studies show that differences can be large depending on the physical properties of the aquifer (Brozovic, Sunding, and Zilberman 2010; Edwards 2016) and uncertainty (Merrill and Guilfoos 2018).

### Land Use Dynamics

Agricultural land can be allocated to irrigated crops (e.g., rice, soybeans, corn) or non-irrigated crops (soybeans, fallow). The study area is divided into *m* sites, each with a fixed land base. At each site *i* and period *t*, land may shift among land uses *j*, denoted by *L*_*ijt*_. The total land area at each site remains constant over time (Eq. 5).

5 $$\mathop{\sum }\limits_{j=1}^{n}{L}_{ijt}=\mathop{\sum }\limits_{j=1}^{n}{L}_{ij0},\,\mathrm{for}\,j=\mathrm{crops},\,\mathrm{fallow}$$

The maximum share of land that can be allocated to crop *j* at site *i* is bounded by historical crop presence. This reflects agronomic and management constraints that prevent the continuous cultivation of the most profitable crops. The upper limit is given by the initial land area in crop *j* at site *i* multiplied by a factor that reflects historical variation in land cover.

### Irrigation and Aquifer Dynamics

Irrigation demand per crop, *wd*_*j*_, is fixed and inelastic, consistent with empirical evidence (Moore et al. 1994; Wang and Segarra 2011). Intra-seasonal shortages are assumed to be resolved through additional well drilling or pump replacement, making the model suitable for long-run analysis of groundwater value.

Each site’s aquifer, *AQ*_*it*_, is modeled independently as a “bathtub.” Modeling the landscape as multiple “bathtub” sites strikes a balance between two unrealistic extremes: an entirely interconnected bathtub in which pumping at any location affects water levels everywhere, and a system of fully isolated sites with no cross-effects from pumping. Actual groundwater flow reflects intricate aquifer geology, which can be examined through simulation but is too complex to incorporate directly into an optimization framework. The stock of water at site *i* in period *t* depends on the previous stock, natural recharge from precipitation and streams, return flows from irrigation, and withdrawals. Groundwater pumping at site *i* in period *t* is denoted *GW*_*it*_. Natural recharge varies by site and is denoted $n{r}_{i}$. The proportion of irrigation water that returns to the aquifer depends on land use (Eq. 6).

6 $$A{Q}_{it}=A{Q}_{i(t-1)}+n{r}_{i}+\mathop{\sum }\limits_{j}^{n}r{f}_{j}w{d}_{j}{L}_{ijt-1}-G{W}_{it}$$

Pumping costs rise with aquifer depth. The groundwater pumping cost at site i in period t, *GC*_*it*_, depends on the cost of lifting an acre-foot of water by one foot, *c*^*p*^, and the depth to the water table, *dp*_*i*_. Depletion of the aquifer stock translates into increased depth to water and, consequently, higher pumping costs. Capital costs, *c*^*c*^, account for well replacement as aquifers decline (Eq. 7).

7 $$G{C}_{it}={c}^{c}+{c}^{p}\left(d{p}_{i}+\frac{(A{Q}_{i0}-A{Q}_{it})}{{\sum }_{j}^{n}{L}_{ij0}}\right)$$

Surface storage is an alternative source of irrigation water. Its capacity depends on the share of land, ${L}_{iRt}$, devoted to storage, rainfall, and runoff capture. Low-end capacity, ${\omega }_{\min }$, reflects rainfall only, while high-end capacity, $({\omega }_{\max }+{\omega }_{\min })$, accounts for both rainfall and recovery of runoff. The acre-feet of water stored in an acre of surface storage (Kovacs et al. 2014) is $({\omega }_{\max }+{\omega }_{\min })-\frac{{\omega }_{\max }}{{\sum }_{j}^{n}{L}_{ij0}}{L}_{iRt}$. We abstract from intra-annual variability in rainfall timing, evaporation, and leakage. Water withdrawn from surface storage, *RW*_*it*_, cannot exceed the water available in storage (Eq. 9).

9 $$R{W}_{it}\le (({\omega }_{\max }+{\omega }_{\min })-\frac{{\omega }_{\max }}{{\sum }_{j}^{n}{\sum }_{k}^{K}{L}_{ijk0}}{L}_{iRt}){L}_{iRt}$$

The water for irrigation is less than or equal to the water taken from wells and surface storage (Eq. 10).

10 $$\mathop{\sum }\limits_{j=1}^{n}w{d}_{j}{L}_{ijt}\le G{W}_{it}+R{W}_{it}$$

### Economic Environment

Crop yields, *y*_*ij*_, are based on site-specific averages, adjusted for long-term productivity growth (Anderson et al. 2009). Crop prices, *pr*_*j*_, and production costs (excluding irrigation), *ca*_*j*_, are constant in real terms. Irrigation costs include surface storage construction and maintenance, *c*^*rw*^, as well as pumping costs for groundwater, *GC*_*it*_, and surface water, *c*^*rw*^. Pumping costs incorporate diesel prices, oil and lubrication, and capital equipment costs.

### Optimization and Solution

The model maximizes discounted profits over a 200-year horizon, approximating an infinite planning horizon. The real discount factor, ${\delta }_{t}$, balances social and market time preferences. Site-level heterogeneity in crop yields, recharge, aquifer depth, and saturated thickness drives variation in model outcomes (Eq. 11).

11 $$\mathop{{\mathrm{max}}}\limits_{{L}_{{ijkt}},R{W}_{{it}},G{W}_{{it}}}:\mathop{\sum }\limits_{t=1}^{T}{\delta }_{t}\left(\mathop{\sum }\limits_{i=1}^{m}\mathop{\sum }\limits_{j=1}^{n}(p{r}_{j}{y}_{ij}-c{a}_{j}){L}_{ijt}-{c}^{r}{L}_{iRt}-{c}^{rw}R{W}_{it}-G{C}_{it}G{W}_{it}\right)$$

The optimization is implemented in GAMS 24.5.6 using the CONOPT solver. Shadow values associated with the aquifer stock constraint (Eq. 6) measure the marginal benefit of conserving groundwater at each site.

## Data

**Fig. 1:**
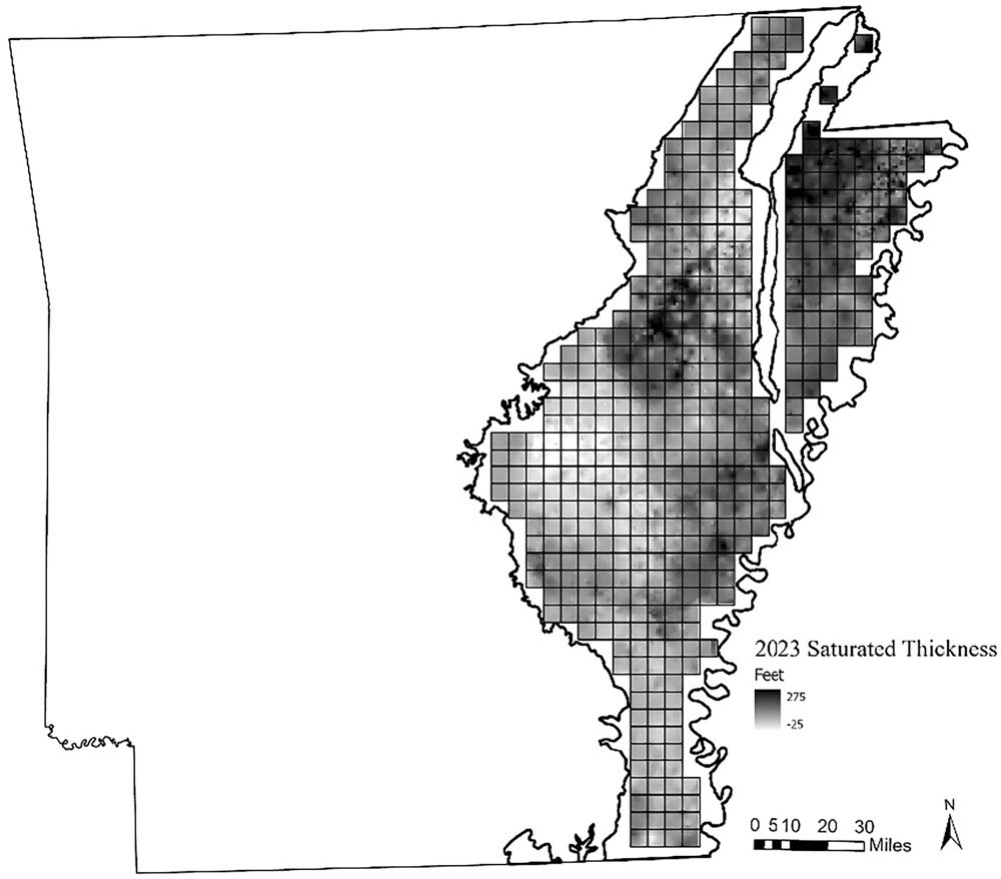
Mississippi River Valley Alluvial aquifer saturated thickness and the study area sites for the hydro-economic model

The study area is defined by the boundary of the Mississippi River Valley Alluvial Aquifer in Arkansas, where groundwater use is concentrated. This region spans 25 counties, which we further divide into 449 sites measuring five miles square, each containing agricultural land (Fig. 1). Our study area and data are drawn from a report about the value of groundwater for the Arkansas Department of Agriculture (Kovacs et al. 2024). Table 7 reports definitions and descriptive statistics of the spatial data across sites, while Table 8 provides the parameters for farm production and the hydrologic model.

Crop areas by site are derived from the 2023 Cropland Data Layer (Johnson and Mueller 2010). Irrigated and non-irrigated soybean areas are distinguished using harvested area data from 2022–2023 (USDA-NASS 2023). Crop yields are based on county-level averages over the past five years (Division of Agriculture 2023), with future yields projected to increase according to long-term crop-specific growth rates from 1867–2009 (Anderson et al. 2009). Production costs and well ownership and maintenance charges are held constant in real terms.

We adopt a 200-year planning horizon to approximate an infinite time frame. Discounting follows the U.S. Office of Management and Budget (2003) guidelines, using the midpoint of the recommended range at 5% to balance social and market time preferences. At this rate, a dollar received two centuries into the future is worth less than one-ten-thousandth of a cent today, effectively placing minimal present value on very distant returns.

### Farm Production

Production cost estimates by crop, excluding irrigation, are taken from the Division of Agriculture (2023). Irrigation requirements represent the average water applied over the growing season, net of rainfall (Division of Agriculture 2023). Crop prices are based on the U.S. baseline outlook projections for agricultural and biofuel markets (FAPRI 2024).

Irrigation costs reflect expenses for labor, fuel, lubricants, poly pipe for border irrigation, and levee gates for rice flood irrigation (McDougall 2015). Although wells in the region were traditionally diesel-powered, electric well use has increased in recent years (USDA-NASS 2014). Groundwater pumping costs are determined by well depth and the associated diesel requirements per acre-foot of water pumped. Additional expenses include the purchase and maintenance of gearheads, power units, and pumps. Surface water pumping costs are based on the diesel needed to move water into and out of surface storage (McDougall 2015). Using the recent average diesel price (EIA 2022), with an additional allowance for oil and lubrication, we find that fuel expenses constitute a major component of total irrigation costs.

Surface storage is replenished through rainfall unless runoff is captured by a tail-water recovery system. Without runoff collection, one acre of surface storage retains about 1.5 acre-feet of water (Young et al. 2004), with a maximum capacity of roughly 8 to 10 acre-feet per acre annually. The main construction costs of surface storage involve soil movement, alongside ongoing expenses for erosion control and pump maintenance. Amortizing these capital and maintenance costs leads to an annualized per-acre charge.

### Aquifer Properties

The depth to the water table and initial saturated thickness of the alluvial aquifer are obtained from the Arkansas Department of Agriculture, Natural Resources Division (ADA DNR 2023). Site-specific values are calculated as spatially weighted averages from interpolated surfaces provided by ADA DNR (2023). Storativity, the proportion of water stored in the aquifer material, is estimated using spatially coarse pilot points from Clark, Westerman, and Fugitt (2013). The initial groundwater volume at each site is the product of site area, saturated thickness, and storativity. Natural recharge is determined using outputs from a soil water balance model (2000–2018), which accounts for net infiltration from precipitation and surface stream interactions (Westenbroek, Nielsen, and Ladd 2021).

## Results

The current cost-share arrangement for on-farm surface storage that the US Department of Agriculture has with farmers in Arkansas is based on USDA-NRCS (2025) payment schedules. Farmers who qualify have 65% of construction costs covered by EQIP. Table 1 shows aquifer thickness changes over time with and without this subsidy. After five years, the difference is small (0.27 feet), but after sixty years, the difference expands to 2.31 feet. Within thirty years, the subsidy increases average aquifer thickness by 10.6%, and within sixty years by 18.7%. The cost of conserving an acre-foot of water falls from $16.12 in year 5 to $5.76 in year 60, while the value of water increases from $16.47 to $28.09. Average benefits exceed average costs in each period, with net benefits rising from $0.35 in year 5 to $22.33 in year 60.

**Table 1:** Benefit and the cost of water conserved from an on-farm surface storage subsidy

Years after present	Average aquifer thickness (feet)		Average cost of the subsidy ($ per acre-foot conserved)	Average benefit ($ per acre-foot)
	No subsidy	On-farm storage subsidy (35% cost covered by the producer)		
5	14.49	14.76	16.12	16.47
30	13.03	14.41	8.94	21.16
60	12.35	14.66	5.76	28.09

Table 2 examines alternative cost-sharing arrangements. As the producer share increases, the average cost of the subsidy declines. Small subsidies concentrate storage investment in the most profitable sites, while larger subsidies extend adoption to less suitable areas, raising average costs. With a high subsidy (producer covers 5%), average costs exceed $23 per acre-foot in year 5, and net benefits are initially negative, turning positive by year 30. By contrast, when producers cover 90%, net benefits are highest, but conservation is minimal.

**Table 2:** Cost of water conserved by intensity of the subsidy for on-farm surface storage

Producer share (% cost covered by the producer)	Average cost of the subsidy ($ per acre-foot conserved)		
	5th year	30th year	60th year
5	23.21	13.54	8.86
35	16.12	8.94	5.76
65	10.44	5.15	3.15
90	7.27	2.61	1.17
	Average benefit ($ per acre-foot)		
	5th year	30th year	60th year
	16.47	21.16	28.09

Site-level results in Table 3 show that with a 35% producer share, more than half of sites (259 of 449) exhibit no change in aquifer volume after 30 years, while 156 sites gain volume and 34 lose volume. Gains occur where surface storage was already prominent; losses occur where no storage existed initially and adoption was limited. Sites with increased aquifer volume had lower saturated thickness without the subsidy but benefited substantially from it. These sites saw profits increase by $188 per acre, but subsidy costs to taxpayers averaged $289 per acre. The cost per acre-foot conserved at these sites ($12.43) exceeded the study-wide average ($8.94).

**Table 3:** Site-level results by water conservation category

	Conservation category (Change in aquifer volume with versus without subsidy)			
	Decrease	No change		Increase
Number of sites	34	259	156	
Average change in the proportion of land in surface storage^a^	0.005	3.0E−04	0.014	
Average proportion of land in surface storage without subsidy^b^	0	0.011	0.048	
Average change in saturated thickness^a^ (feet)	−2.22	0.00	11.67	
Saturated thickness without subsidy^b^ (feet)	13.88	21.15	9.30	
Average change in profits per acre^a^ ($/acre)	19.12	12.76	188.86	
Average cost of the subsidy per acre^a^ ($/acre)	18.02	13.82	289.41	
Average cost of the subsidy^a^ ($ per acre-foot conserved)	-	-	12.43	
Average benefit^b^ ($ per acre-foot)	27.9	20.6	20.6	
Average benefit minus cost of subsidy^a^ ($ per acre-foot)	-	-	8.16	

^a^This is with versus without the subsidy of 35% cost covered by the producer for the 30th year. ^2^ This is for the 30th year and no subsidy for surface storages

Regression results in Table 4 show that land allocation and crop yields strongly shape storage adoption and conservation benefits. Higher initial shares of irrigated rice and higher yields of corn and rice increased storage adoption, while higher shares and yields of soybeans and cotton reduced it. Deeper initial aquifers also encouraged storage adoption, while higher natural recharge discouraged it. The benefit per acre-foot of water conserved was greater in sites with higher rice, corn, and soybean shares and yields, but lower where aquifers were deeper, thicker, or more naturally recharged. Results were similar in years 5 and 30.

**Table 4:** Regression results for site level proportion of land in surface storage and the benefit of groundwater

Independent variable	Dependent variable		
	Proportion of site in surface storage^a^	Benefit in the 30th year^a^ ($ acre-foot)	Benefit in the fifth year^b^ ($ acre-foot)
Initial proportion of site			
Irrigated corn	−0.041 (0.05)^b^	20.81 (0.01)^a^	6.84 (0.09)^c^
Irrigated rice	0.078 (0.00)^a^	55.28 (0.00)^a^	23.15 (0.00)^a^
Irrigated soybean	−0.032 (0.06)^c^	16.24 (0.01)^a^	0.799 (0.81)
Dryland soybean	−0.011 (0.56)	−0.868 (0.92)	−19.87 (0.00)^a^
Irrigated cotton	3.94E−05 (0.21)	0.019 (0.16)	−0.005 (0.44)
Yield of the crop			
Irrigated corn	0.001 (0.02)^b^	0.251 (0.00)^a^	0.166 (0.00)^a^
Irrigated rice	2.72E−05 (0.00)^a^	0.003 (0.32)	0.008 (0.00)^a^
Irrigated soybean	−0.001 (0.00)^a^	−0.154 (0.18)	−0.052 (0.29)
Dryland soybean	−0.001 (0.00)^a^	0.135 (0.28)	−0.522 (0.00)^a^
Irrigated cotton	−5.1E-05 (0.09)^c^	0.021 (0.07)^c^	−0.002 (0.63)
Initial depth to aquifer	0.0003 (0.00)^a^	−0.189 (0.00)^a^	−0.208 (0.00)^a^
Initial saturated thickness of aquifer	−5.5E−05 (0.15)	−0.047 (0.02)^b^	−0.044 (0.00)^a^
Natural recharge	−0.115 (0.00)^a^	−27.22 (0.00)^a^	−6.76 (0.01)^a^
Adjusted R^2^	0.53	0.29	0.63

^a^This is for the 30th year and no subsidy for surface storages. ^2^ This is for the 5th year and no subsidy for surface storages. Number of observations is 449. *p*-value in parentheses. Standard errors calculated through 5000 bootstrap replications. ^a^significant at 1% level; ^b^significant at 5% level; ^c^significant at 10% level

Table 5 shows that under the subsidy policy, sites with higher rice yields, greater initial depth to the aquifer, and larger saturated thickness allocated more land to storage and increased aquifer thickness. By contrast, higher yields of soybeans and cotton were associated with declines in saturated thickness, indicating less effective conservation.

**Table 5:** Regression results for site-level change in the proportion of land in surface storage and the change in saturated thickness with versus without subsidy

Independent variable	Dependent variable^a^	
	Change in the proportion of site in surface storage	Change in saturated thickness (feet)
Initial proportion of site		
Irrigated corn	0.0002 (0.98)	−2.76 (0.36)
Irrigated rice	0.006 (0.38)	1.89 (0.47)
Irrigated soybean	−0.002 (0.77)	−0.909 (0.66)
Dryland soybean	0.005 (0.48)	−6.67 (0.02)^b^
Irrigated cotton	1.32E−05 (0.24)	0.007 (0.12)
Yield of the crop		
Irrigated corn	−4.2E−05 (0.60)	0.033 (0.20)
Irrigated rice	8.31E−06 (0.00)^a^	0.005 (0.00)^a^
Irrigated soybean	−0.0002 (0.14)	−0.213 (0.00)^a^
Dryland soybean	2.34E−05 (0.77)	−0.122 (0.00)^a^
Irrigated cotton	−4.23E−06 (0.72)	−0.012 (0.00)^a^
Initial depth to aquifer	0.0004 (0.00)^a^	0.212 (0.00)^a^
Initial saturated thickness of aquifer	5.46E−05 (0.00)^a^	0.041 (0.00)^a^
Natural recharge	0.009 (0.21)	3.23 (0.13)
Adjusted R^2^	0.37	0.61

^a^ The dependent variable is the difference in the 30th year with the subsidy minus without the subsidy. The subsidy is the of 65% cost covered by the government. Note: Number of observations is 449. *p*-value in parentheses. Standard errors calculated through 5000 bootstrap replications. ^a^significant at 1% level; ^b^significant at 5% level; ^c^significant at 10% level

Finally, Table 6 compares determinants of subsidy cost and net benefit. Conservation costs were higher at sites with high dryland soybean and cotton yields, but lower where aquifers were deeper, thicker, or better recharged. Net benefits were greatest at sites with high rice yields and low dryland soybean yields, as well as where aquifers were shallow and recharge was high.

**Table 6:** Regression results for the site-level benefit, cost, and the benefit minus cost of the subsidy

Independent variable	Dependent variable		
	Benefit in the 30th year^1^ ($ acre-foot)	Cost of the subsidy^2^ ($ per acre-foot conserved)	Benefit minus cost of subsidy^2^ ($ per acre-foot)
Initial proportion of site			
Irrigated corn	20.81 (0.01)^a^	6.69 (0.93)	-42.43 (0.57)
Irrigated rice	55.28 (0.00)^a^	5.87 (0.94)	−26.98 (0.74)
Irrigated soybean	16.24 (0.01)^a^	−10.46 (0.89)	−33.57 (0.67)
Dryland soybean	−0.868 (0.92)	−39.43 (0.64)	−15.69 (0.85)
Irrigated cotton	0.019 (0.16)	−0.006 (0.92)	−0.026 (0.62)
Yield of the crop			
Irrigated corn	0.251 (0.00)^a^	0.238 (0.11)	0.0281 (0.85)
Irrigated rice	0.003 (0.32)	−0.007 (0.12)	0.010 (0.02)^b^
Irrigated soybean	−0.154 (0.18)	0.185 (0.55)	−0.388 (0.19)
Dryland soybean	0.135 (0.28)	0.621 (0.06)^b^	−0.730 (0.02)^b^
Irrigated cotton	0.021 (0.07)^c^	0.067 (0.09)^b^	−0.048 (0.23)
Initial depth to aquifer	−0.189 (0.00)^a^	−0.278 (0.00)^a^	−0.084 (0.02)^b^
Initial saturated thickness of aquifer	−0.047 (0.02)^b^	−0.073 (0.02)^b^	0.010 (0.72)
Natural recharge	−27.22 (0.00)^a^	−12.99 (0.01)^a^	13.82 (0.00)^a^
Adjusted R^2^	0.29	0.39	0.16
Number of observations	449	156	156

^1^ This is for the 30th year and no subsidy for surface storages. ^2^ This is for the 30th year and 65% cost covered by the government. Number of observations is 156. *p*-value in parentheses. Standard errors calculated through 5000 bootstrap replications. ^a^significant at 1% level; ^b^significant at 5% level; ^c^significant at 10% level

## Discussion

Our results indicate that cost-share incentive programs for on-farm surface storage in Arkansas promote long-run aquifer conservation and yields positive social net benefits, but the timing and distribution of benefits matter for policy design. While aggregate conservation effects are modest in the first decade, they compound over time, producing much larger gains after 30–60 years. This lag may reduce the political appeal of such subsidies, particularly for policymakers with high discount rates, but underscores their long-term value. Similar long-run versus short-run trade-offs have been observed in Spain, where managed aquifer recharge projects demonstrated increasing benefits only after decades of accumulation (Custodio et al. 2016).

The analysis of alternative cost-sharing levels highlights a trade-off between scale and efficiency. Smaller subsidies (higher producer shares) channel investment into the most suitable sites, minimizing average conservation costs but limiting the overall volume of water conserved. Larger subsidies expand adoption geographically but raise average costs, particularly in areas less well-suited for storage. These findings resonate with international scholarship showing that subsidy design critically shapes both cost-effectiveness and equity in conservation programs (OECD 2017). In India, for example, generous electricity subsidies promoted widespread irrigation pumping but substantially reduced cost-effectiveness and contributed to rapid aquifer depletion (Varady et al. 2013; Mukherji 2022).

Site-level heterogeneity is central: surface storage adoption and conservation outcomes were most effective in areas already oriented toward rice production, where water demand is high and storage complements existing irrigation practices. Conversely, areas dominated by soybeans and cotton, which are less water-intensive, saw little or negative conservation effects. This mirrors evidence from Mexico, where government subsidies for irrigation infrastructure had the strongest conservation effects in water-intensive maize regions but were less effective in areas with drought-tolerant crops (Scott & Shah 2007; Hoogesteger, and Wester 2017).

Our results also demonstrate that natural aquifer characteristics condition policy effectiveness. Sites with deeper water tables adopted more storage under subsidies, likely due to higher pumping costs, while high natural recharge reduced the marginal value of storage. This parallels experiences in Australia’s Murray–Darling Basin, where groundwater trading and conservation incentives were most effective in over-extracted, high-cost pumping zones (Grafton et al. 2014). Similarly, in Spain and North Africa, subsidy programs for aquifer recharge and storage were most cost-effective in areas with shallow water tables and high irrigation demand (Giordano 2009).

## Conclusion

This study demonstrates how applying the net benefit criterion to groundwater conservation policy provides a clear framework for evaluating the cost-effectiveness of subsidies at the site level. Using the case of subsidized on-farm surface storage in the Lower Mississippi River Basin of Arkansas, we show that current cost-sharing levels (65% government coverage) generate positive long-run net benefits, with the average benefit of conserved water ($21/acre-foot) exceeding its cost ($8.94/acre-foot) after 30 years. While lower subsidies reduce the cost per unit of conservation, they also result in less overall aquifer preservation, highlighting the trade-off between efficiency and conservation scale. Site-specific factors strongly influence these outcomes: high yields of water-intensive crops such as rice and corn increase benefits, while high yields of less water-intensive crops such as soybeans and cotton raise costs.

Our results suggest that subsidies for surface storage are most effective in locations with abundant natural recharge, shallow aquifers, and intensive rice cultivation, where both conservation and net benefits are maximized. Conversely, regions dominated by non-irrigated crops or limited by recharge potential are less suitable for such subsidies. Future extensions could incorporate climate variability, competitive extraction dynamics, or alternative conservation strategies such as groundwater extraction fees, efficiency-based irrigation subsidies, or pumping caps. Comparative analysis of these approaches would further clarify how policy design can align economic incentives with sustainable groundwater use.

## Data Availability

Data will be available upon request.

## Appendix

Tables 7 and 8

**Table 7:** Descriptive statistics of the spatial data across sites of the study area

Definition	Average	Std. Dev.
Initial acres of rice	2212.95	2051.31
Initial acres of irrigated corn	1300.23	1035.61
Initial acres of irrigated soybean	5033.71	2318.52
Initial acres of dry land soybean	847.83	767.99
Initial acres of cotton	793.53	1633.05
Annual rice yield (pounds per acre)	7450.62	195.31
Annual corn yield (bushel per acre)	173.58	10.10
Annual irrigated soybean yield (bushel per acre)^1^	50.82	6.49
Annual dry land soybean yield (bushel per acre)^1^	35	7.58
Annual cotton yield (pounds per acre)	1234.51	57.04
Depth to water (feet)	43.45	28.78
Initial aquifer thickness (feet)	97.96	41.04
Annual natural recharge of the aquifer per acre per year (acre-feet)	0.43	0.12

Note: Number of all sites is 449. ^1^ The mean and the standard deviation of the county yields come from the 31 counties in the study area

**Table 8:** Value of economic and irrigation model parameters

Definition	Value
Price of rice ($/pound)	0.18
Price of corn ($/bushel)	5.75
Price of soybeans ($/bushel)	13.60
Price of cotton ($/pound)	0.85
Annual increase in rice yield (%)	1.6
Annual increase in corn yield (%)	1.34
Annual increase in soybean yield (%)	1.58
Annual increase in cotton yield (%)	1.17
Annual production cost of rice ($/acre)	796
Annual production cost of irrigated corn ($/acre)	856
Annual production cost of irrigated soybean ($/acre)	502
Annual production cost of dry land soybean ($/acre)	454
Annual maintenance cost of fallow ($/acre)	22
Annual irrigation per acre of rice (acre-foot)	2.7
Annual irrigation per acre of corn (acre-foot)	1.17
Annual irrigation per acre of full-season soybean (acre-foot)	1.0
Proportion of applied water to rice that returns to aquifer	0.15
Proportion of applied water to corn that returns to aquifer	0.2
Proportion of applied water to full-season soybean that returns to aquifer	0.2
Proportion of applied water to cotton that returns to aquifer	0.2
Unitless measure of the proportion of water stored in a cubic meter of soil (Storativity)	0.2
Rainfall recovery per acre of surface storage (acre-feet)	1.5
Tailwater recovery per acre of surface storage (acre-feet)	7.5
Baseline annual per acre cost of surface storage ($/acre)^a^	377
Cost to re-lift an acre-foot to and from the surface storage ($/acre-foot)	22.62
Cost to raise acre-foot of water by one foot ($)	0.547
Discount factor	0.95

^a^ This is the annual amortized construction cost and maintenance cost over thirty years for each acre of surface storage
